# Conservation implications of mapping the potential distribution of an Ethiopian endemic versatile medicinal plant, *Echinops kebericho* Mesfin

**DOI:** 10.1002/ece3.10061

**Published:** 2023-05-07

**Authors:** Bedilu Tafesse, Tamrat Bekele, Sebsebe Demissew, Bikila Warkineh Dullo, Sileshi Nemomissa, Desalegn Chala

**Affiliations:** ^1^ Department of Plant Biology and Biodiversity Management, College of Natural Sciences Addis Ababa University Addis Ababa Ethiopia; ^2^ Natural History Museum University of Oslo Oslo Norway; ^3^ Center for Biodiversity Dynamics in a Changing World (BIOCHANGE), Department of Biology Aarhus University Aarhus C Denmark; ^4^ Section for Ecoinformatics and Biodiversity, Department of Biology Aarhus University Aarhus C Denmark

**Keywords:** climate change, conservation, Ethiopia, MaxEnt, reintroduction, species distribution modeling, threatened species

## Abstract

*Echinops kebericho* is a narrow‐range multipurpose medicinal plant confined to Ethiopia. Intense land use change and overharvesting for traditional medicine have resulted in narrow distributions of its populations. It is a threatened species with a decreasing population trend. This study aims to map its potential distribution, which is key to guide conservation efforts and sustainable use. We modeled the potential distribution of *E. kebercho* using the maximum entropy model (MaxEnt) employing 11 less correlated predictor variables by calibrating the model at two complexity levels and replicating each model 10 times using a cross validation technique. We projected the models into the whole of Ethiopia and produced binary presence–absence maps by classifying the average map from both complexity levels applying three threshold criteria and ensembling the resulting maps into one for the final result. We mapped suitable habitat predicted with high certainty and identified local districts where *E. kebericho* can be cultivated or introduced to enhance its conservation. We estimated that *E.kebercho* has about 137,925 km^2^ of suitable habitat, mainly concentrated in the western highlands of the Ethiopian mountains. Our models at both complexity levels had high average performances, AUC values of 0.925 for the complex model and 0.907 for the simpler model. The variations in performance among the 10 model replicates were not remarkable, an AUC standard deviation of 0.040 for complex and 0.046 for simple model. Although *E. kebericho* is locally confined, our models predicted that it has a remarkably wider potential distribution area. We recommend introducing *E. kebericho* to these areas to improve its conservation status and tap its multiple benefits on a sustainable basis. Locally confined threatened plants and animals likely have wider potential distributions than their actual distributions and thus similar methodology can be applied for their conservation.

## Graphical abstract

*Echinops kebericho* is a narrow‐range multipurpose medicinal plant confined to Ethiopia and known for its efficacy to cure several disease such as epilepsy, epistaxis, and atrophy. Extracts from *E. kebercho* have antibacterial and anti‐Leishmanial activities as well as several agricultural and veterinary applications. Currently, intense land use and overharvesting for traditional medicine have resulted in its narrow distributions, and *E. kebercho* is among the medicinal plants of Ethiopia with a decreasing population trend.

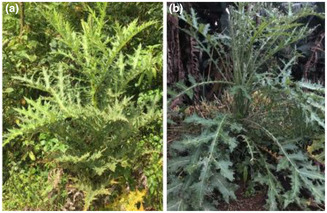

## 1 INTRODUCTION

Biodiversity loss is taking place at an alarming rate, and its concern is currently on top of the global agenda. This is particularly the case in the less developed tropical countries where the majority of the population depends on nature to meet their basic needs (Fedele et al., 2021). Causes of biodiversity loss include land use change, habitat fragmentation, overexploitation, pollution, invasive species, and climate change (Fahrig, 2003; Newbold, 2018; Waldron et al., 2017; Watson et al., 2005; Wilson et al., 2016). Several species are facing extinction because of these anthropogenic activities (Tilman et al., 2017).

Anthropogenic‐driven extinction rate during the past 500 years is estimated to be up to 50‐fold higher than the natural rate (Barnosky et al., 2011). Over the past 10,000 years, 50% of the ice‐free terrestrial surface has been modified by humans (Lambin et al., 2003). This change in land use has highly affected biodiversity, ecosystem functions, and service provisions (García‐Vega & Newbold, 2020; Koellner et al., 2013; Wilson et al., 2016). Land cover and land use change affect biodiversity at ecosystem, species, population, and gene levels (de Baan et al., 2013; de Groot et al., 2012).

Exploitation of resources at a faster rate than their natural regeneration is becoming the most common challenge of the Anthropocene (de Souza & Prevedello, 2020; Hoffmann et al., 2014). Overexploitation for different purposes such as for household consumption, trade, recreation, and subsistence is one of the factors behind quite large number of the species that are currently listed as threatened or near‐threatened in the IUCN red list (Maxwell et al., 2016). Overexploitation decreases population density directly by reducing the number of individuals (Burgess et al., 2017), and it may have indirect impacts by decreasing reproduction (Pillay et al., 2018). It may reduce long‐term population viability for these reasons resulting in extinction of populations at different scales (Mora et al., 2007).

Climate change is another factor that results in biodiversity loss causing species range shifts (Scarano & Ceotto, 2015) and redistribution of life on earth (Pecl et al., 2017). Different species have different rate of range shifts in response to climate change, and the majority of the species lag behind the shifting climatic zones (Lenoir et al., 2020). This is affecting species assemblage at local and global scales jeopardizing species interactions and deteriorated ecosystem functions and its ability to provide societies with goods and services (Pecl et al., 2017). Though not well studied, the impact of climate change in Africa may be higher when compared with the other parts of the world (Chala et al., 2016; Davis et al., 2019; Malhi et al., 2013; Peters et al., 2019).

Human activity is expected to continue worsening and posing the major threats to African biodiversity (Midgley & Bond, 2015; Sala et al., 2000). Projected population rise and economic growth are expected to exacerbate the loss of biodiversity and put many more species at risk of extinction worldwide (Tilman et al., 2017). Recent studies over a wide geographic scale show that a third of the tropical African flora is potentially threatened with extinction, whereas another one third of the species are likely rare, potentially becoming threatened in the near future (Stévart et al., 2019). This calls for the need of immediate intervention.

To mitigate or minimize threats to global biodiversity, greater conservation efforts, as well as dedicated measures such as changes in agricultural practices and better land‐use plannings are highly required (Balmford & Bond, 2005; IPCC, 2014). The International Union for the Conservation of Nature's Red List of Threatened Species predicts that 4161 different kinds of species are threatened by climate change, 33% are at risk from climate change‐induced habitat shifts and alteration, and 29% are at risk due to drought (Warmenbol & Smith, 2018). Species that need high conservation priority are identified based on the level of threats that is posed on them (Wilson et al., 2016).

Approximately 80% of the Ethiopian people relies on traditional medicines to treat various human and livestock diseases (IBC, 2005). In Ethiopia, medicinal plants play significant role in prevention and treatments of several diseases (Flatie et al., 2009; Gedif & Hahn, 2003). There are several reasons for the majority of Ethiopians to continue relying on traditional medicines. Limited health care coverage, unaffordable price of modern medicines and a belief that herbal medicines have better efficacy are usually presented as reasons (Andarge et al., 2015; Kidane et al., 2018; Yirga & Zeraburk, 2011). In spite of this, medicinal plant biodiversity and knowledge associated with their use and conservation is declining and disappearing at an alarming rate (Andarge et al., 2015). The problem encompasses medicinal plants that are endemic to the country and locally confined in their distributions (IBC, 2005).

**FIGURE 1:**
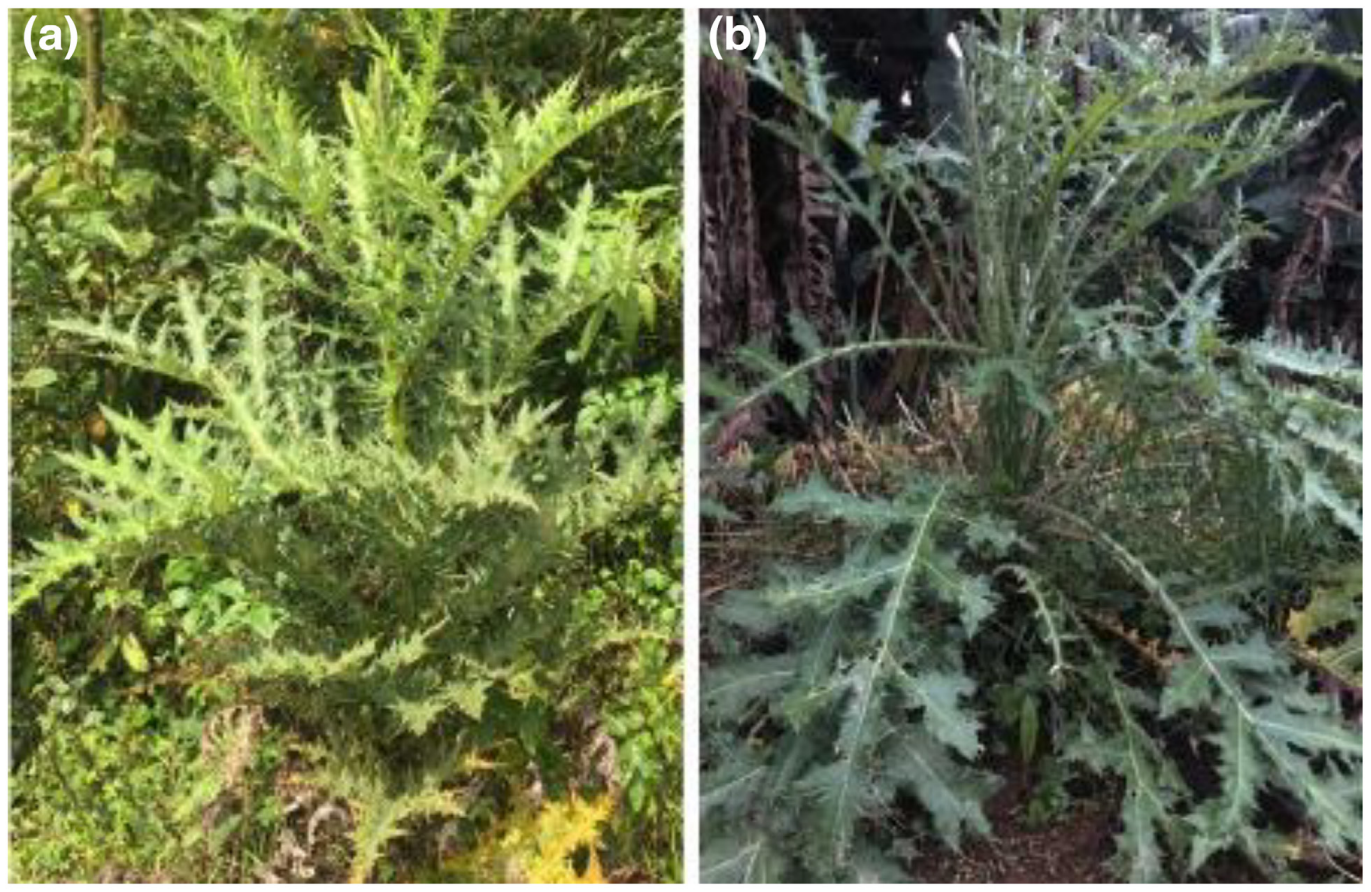
*Echinops kebericho* (a) growing on a grazing land, (b) growing in home garden. Photos: Bedilu Tafesse.

*Echinops kebercho* is a multipurpose traditional medicinal plant confined to Ethiopia in distribution (Tadesse, 2004; Figure 1). It is used to treat various infectious diseases, and bioactive extracts of *E. kebericho* have antibacterial activities (Deyno et al., 2021; Fikadu & Melesse, 2014; Tariku & Kebede, 2011; Toma et al., 2015). The extracts of its rootstock cure epilepsy, epistaxis, atrophy, and sudden sickness (Maryo et al., 2015), exhibit high anti‐Leishmanial activity (Tariku et al., 2011), and can be used to control insect pests of medical, veterinary, and agricultural values (Hussien et al., 2011). The rootstock of this species is sold in markets in different parts of Ethiopia (Regassa, 2013). Overharvesting and land use change have reduced local populations, inducing local extinctions (Fikadu & Melesse, 2014). The use of roots for traditional medicine is the major cause of the decline of populations compared to the uses of other parts of the plant (Aschale et al., 2018; Gebeyehu et al., 2014; Ragunathan & Abay, 2009). Deforestation (Vivero et al., 2006) and agricultural expansion (Behailu & Temesgen, 2017) are also resulting in diminishing status of local populations of medicinal plants as a whole. This species has been assessed as near threatened (Darbyshire et al., 2021) and was also undergoing local extinctions in its native range.

Mapping the distributions of potential suitable habitats of *E. kebericho* is important for its conservation. Endemic and threatened plant species are critical components of plant biodiversity, and their long‐term survival requires immediate human intervention (Bahadur et al., 2015). Predicting and mapping potential suitable habitat for threatened and endangered species is crucial for tracking and conserving declining native populations in their natural habitat (Balmford & Bond, 2005). However, data on the distribution of threatened and endangered species is most often missing (Elith et al., 2006; Engler et al., 2004).

Species distribution modeling tools are gaining popularity in ecology and are being used in many ecological applications (Elith et al., 2006; Peterson, 2008). It is used in conservation biology (e.g., Gebremedhin et al., 2021; Warren et al., 2014), epidemiology (Cardoso‐Leite et al., 2014), invasion biology (Palaoro et al., 2013) and in several fields of biology such as evolution (Chala et al., 2016, 2019; Schmidt‐Lebuhn et al., 2015). It is used to understand the relationship between species and their environment and to predict their actual and potential distributions. Various species distribution modeling methods are available with different performances and data type requirements (Elith et al., 2006; Guisan et al., 2007; Guisan & Zimmermann, 2000; Kumar & Stohlgren, 2009). MaxEnt is among the top performing algorithms, and it uses presence‐only occurrence data (Phillips & Dudík, 2008).

For rare and endangered plant species, relatively few predictive models have been used (Engler et al., 2004). Here we model and quantify the potential suitable habitats of *E. keberich*o in Ethiopia and map areas where it can be cultivated for conservation and medicinal uses.

## 2 METHODS

### 2.1 Occurrence data

**FIGURE 2:**
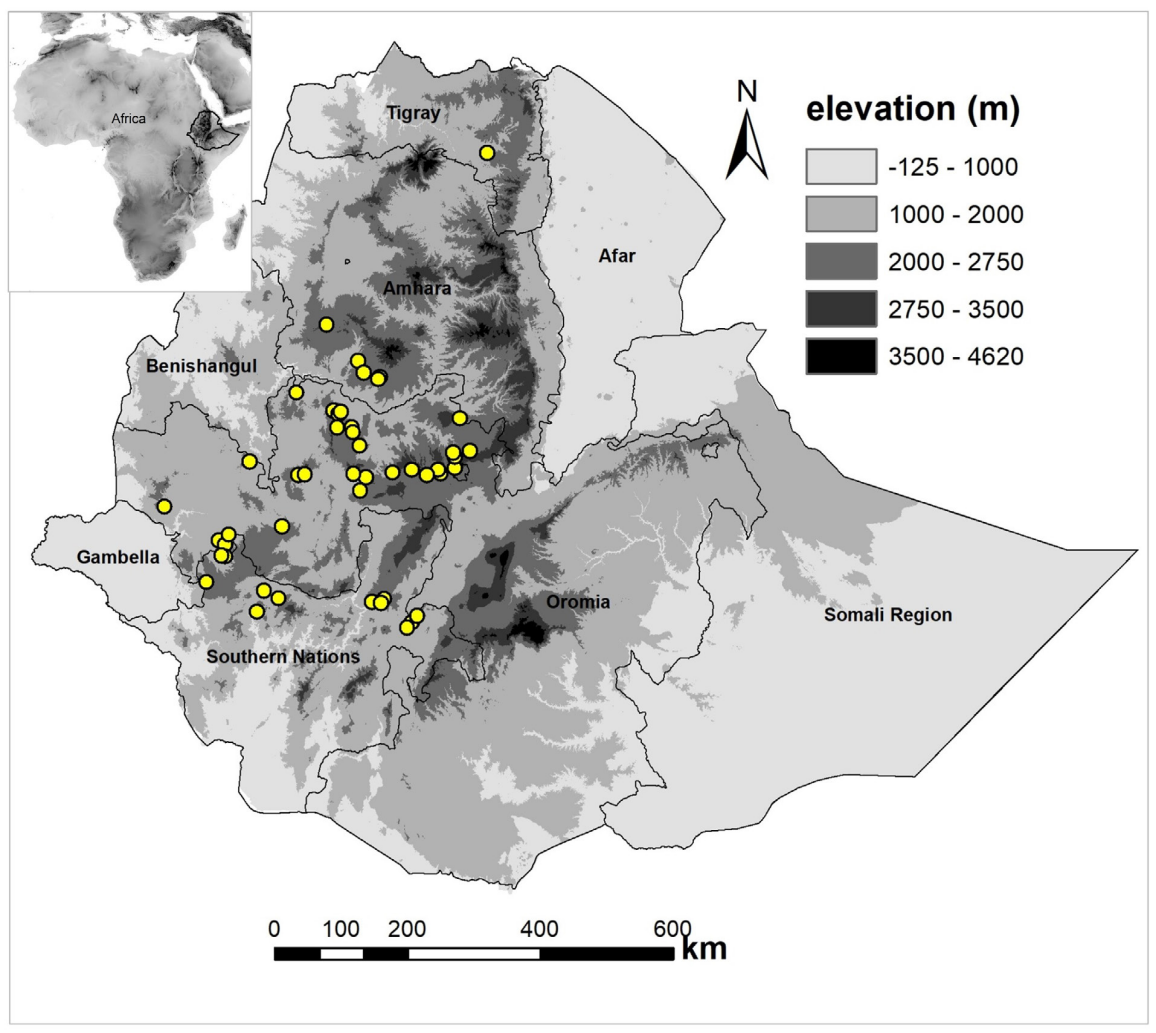
Map of study area; occurrence points of *Echnops kebericho* are presented in yellow points.

We used 49 occurrence data from three data sources: from the National Herbarium of Ethiopia (11), from our own collections (35) and three points from Global Biodiversity Information Facility (GBIF.org, 2021; Figure 2). We filtered these points using a raster layer of 1 × 1 km grid resolution and made sure that none of them are duplicates. We additionally generated 10,000 random points in the study area to serve as random pseudoabsence points (see the detail under Section 2.3).

### 2.2 Environmental variables

A total of 21 predictor variables (19 bioclimatic variables plus topographic position index and solar radiation index) were used. The bioclimatic variables were obtained from the WorldClim database (Fick & Hijmans, 2017) at 30 arc seconds (~1 km) resolution. The topographic data were acquired from www.gscloud.cn (Aster DEM; space‐borne thermal emission and reflection radiometer Digital Elevation Model). The solar radiation (SR) data were taken from https://www.worldclim.org/. Topographic variables have a substantial impact on natural resource distribution by restricting hydrological cycle and soil moisture content (Loritz et al., 2019; Mukherjee et al., 2013). Solar radiation is hypothetically proportional to the amount of direct solar radiation reaching the earth's surface varying along altitude, aspect, slope, and latitude (Keating et al., 2007). Prior to running the models, the multicollinearity of the predictor variables was checked by using pairwise Pearson's correlations. From those variables with a correlation of *r* ≥ .7, only one variable was selected based on assumed biological importance (Chala et al., 2016; Dormann et al., 2007; Kumar & Stohlgren, 2009; Yang et al., 2013; Table S1). For the final model runs, we retained 11 predictor variables: Bio‐1 (annual mean temperature), Bio‐3 (isothermally), Bio‐4 (temperature seasonality), Bio‐7 (temperature annual range), Bio‐11 (mean temperature of the coldest quarter), Bio‐15 (precipitation seasonality), Bio‐17 (precipitation of the driest quarter), Bio‐18 (precipitation of the warmest quarter), Bio‐19 (precipitation of the coldest quarter), solar radiation index, and topographic position index.

### 2.3 Modeling procedure

We used Maximum Entropy (MaxEnt) algorithm version 3.4.1 (Phillips et al., 2006) to model the potential distribution of *E. kebericho*. The MaxEnt algorithm is one of the best performing algorithms and predicts the possible ranges of species by determining the distribution of maximum entropy (Peterson, 2008; Phillips et al., 2006). MaxEnt is especially useful for species with limited presence data where false absence in surveys poses a significant risk (Bosso et al., 2013; Vasconcelos et al., 2012). However, its default setting is complex and its performance may vary with model complexity levels (Halvorsen et al., 2016). Thus, we run the model at two complexity levels (one simple and one complex) to account for this. The complex model was built by setting the regularization multiplier to default (which is 1) and the simpler version built by setting regularization multiplier to 8 (Chala et al., 2016). We used the selected environmental variables along with pseudoabsence and presence points in the model by setting the maximum iterations to 500. We run both the simple and complex models 10 times by sub setting the occurrence data and the randomly generated absence points to 10 using cross validation approach. While 9/10 of the subsets of the occurrence and pseudoabsence datasets were used for model training, the remaining 1/10 was used for model validation at each turn. We used the average result from these 10 runs for reporting. We validated all model runs using the area under ROC curve (AUC). From the final map, we produced binary presence/absence (suitable/unsuitable) maps using three thresholding techniques: 10% presence or by omitting the least probabilities values at 10% of the presence points; maximum sum training sensitivity plus specificity or the probability values that maximizes the sum of the fraction of correctly predicted presences (sensitivity) and the fraction of correctly predicted absences (specificity); and maximum sum test sensitivity plus specificity or a maximum specificity and sensitivity computed on the test data. This means we produced six binary maps as the three thresholding criteria are applied for both model complexity levels. We overlaid the six binary maps and further classified them into three classes based on the agreements in the number of pixels predicting habitat suitability (Chala et al., 2016): unsuitable—when 0–30% (up to one in six) maps predicts habitat suitability; uncertain—when 30%–60% (two to three) maps predicts suitability; and suitable with certainty—when >60% (four to six) binary maps predict suitability (Figure 6). A Jackknife analysis was used to determine variable importance's. We overlaid the areas predicted with high certainty with local districts to identify areas where *E. kebericho* can be cultivated for medicinal uses and also to provide practical guide for conservation scientists.

## 3 RESULTS

### 3.1 Model performance and variable importance

Both simple and complex models had high performances, AUC values of 0.907 and 0.925, respectively. The AUC standard deviation among the 10 models runs was 0.040 for the complex model and 0.046 for the simple model.

The variable importance computed on a test data indicates that the distribution of *E. kebericho* was mainly influenced by solar radiation, mean temperature of the coldest quarter, precipitation of the coldest quarter, and annual mean temperature contributing 42.5%, 30%, 9.9%, and 6.4%, respectively, to the Maxent model regularization 1 (Table 1). The contributions of these variables to the simple model is a bit different in magnitude but of the same respective order of importance. These variables also have higher permutation importance.

**TABLE 1:** Estimate of average contribution and permutation importance of the environmental variables used in MaxEnt modeling of *Echnops kebericho* (%c = percent contribution and PI = permutation importance) under regularization multiplier 1 and 8.

Variables	Regularization multiplier 1.		Regularization multiplier 8.	
	%C	PI	%C	PI
Annual mean temperature (Bio‐1)	*6.4*	9.2	*5.5*	*14.8*
Isothermality (Bio‐3)	*1.3*	0.9	*3*	*3.4*
Temperature seasonality (Bio‐4)	*1*	17.5	*2.1*	*0.8*
Temperature annual range (Bio‐07)	*0.1*	0.9	*0*	*0*
Mean temperature of coldest quarter (Bio‐11)	*30*	44.3	*17.1*	*1.9*
Precipitation seasonality (Bio‐15)	*0.9*	1.3	*0*	*0*
Precipitation of driest quarter (Bio‐17)	*2.7*	0.1	*0*	*0*
Precipitation of warmest quarter (Bio‐18)	*2*	1.4	*0.2*	*0*
Precipitation of coldest quarter (Bio‐19)	*9.9*	2.8	*8.2*	*5.6*
Solar radiation (Sri)	*42.5*	18.4	*63.3*	*71.5*
Topographic index (tpi)	*6.4*	9.2	*0.7*	*2*

### 3.2 Potential habitat distribution of *E. kebericho*

**FIGURE 3:**
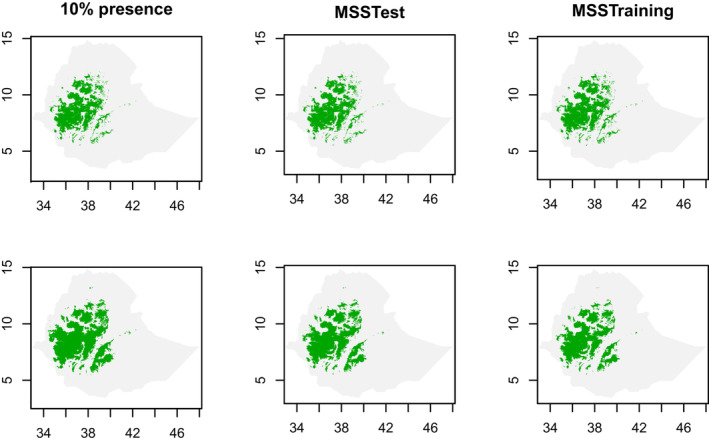
Binary habitat suitability maps produced using two model complexity levels (regularization multiplier 1—upper row and regularization multiplier 8—lower row) and three threshold criteria (10% presence—at 10% training data omission; MSSTest—maximum sum sensitivity and specificity computed on test data; MSSTrain—maximum sum sensitivity and specificity computed on training data).

**FIGURE 4:**
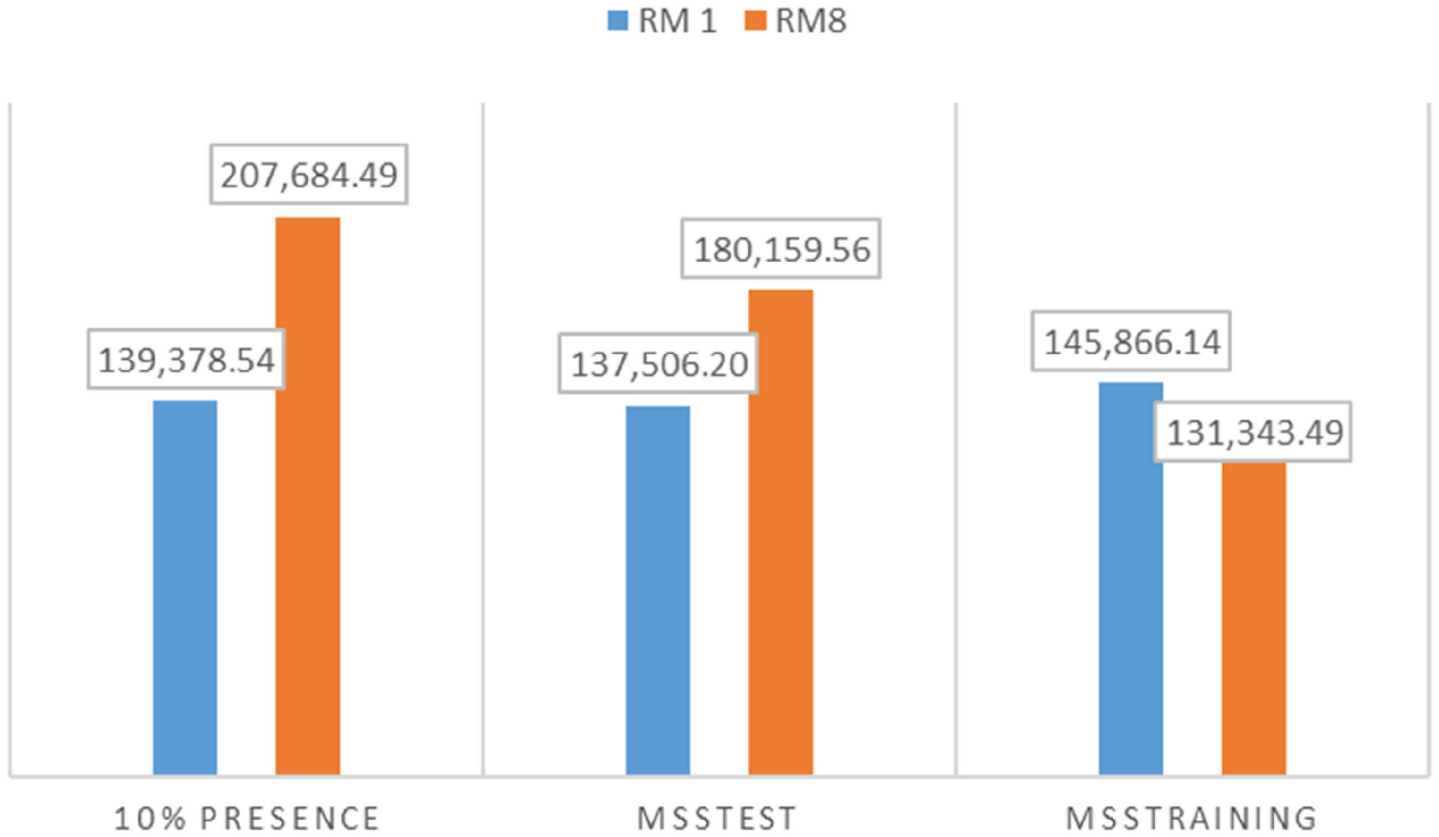
Habitat suitability at three thresholds using two model complexity levels (regularization multipliers 1 (RM1) and 8 (RM8)). MSSTest—maximum sum sensitivity and specificity computed on test data, MSSTraining—Maximum training sensitivity plus specificity, 10% presence–10 percentile training presence.

**FIGURE 5:**
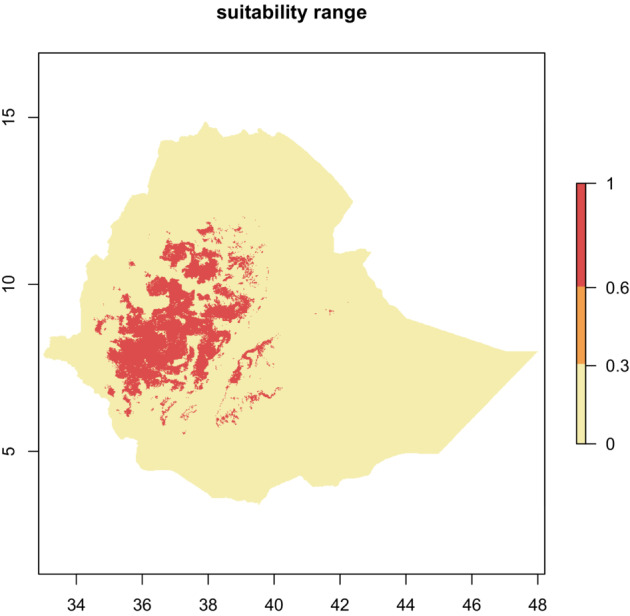
Current habitat suitability of *Echinops kebericho* produced by combining six binary maps produced employing two model complexity levels of MaxEnt, and three cutoff threshold criteria (2 * 3). 0–0.3 shows where none or only one of the six binary maps predicts suitability; 0.3–06 shows where 2–3 of the six binary maps predict suitability and 0.6–1 shows where 4–6 of the six binary maps predict habitat suitability.

Models of both complexity levels at all the three threshold probability cut‐off points consistently showed that the potential suitable habitat of *E. kebericho* is found on the western massifs of Ethiopian highlands (Figures 3 and 5). The potential distribution patterns did not show remarkable variations between model complexity levels and among the cutoff thresholds (Figure 3). However, the simpler model captured a broader distribution area except when the MSSTest threshold is used (Figures 3 and 4).

**FIGURE 6:**
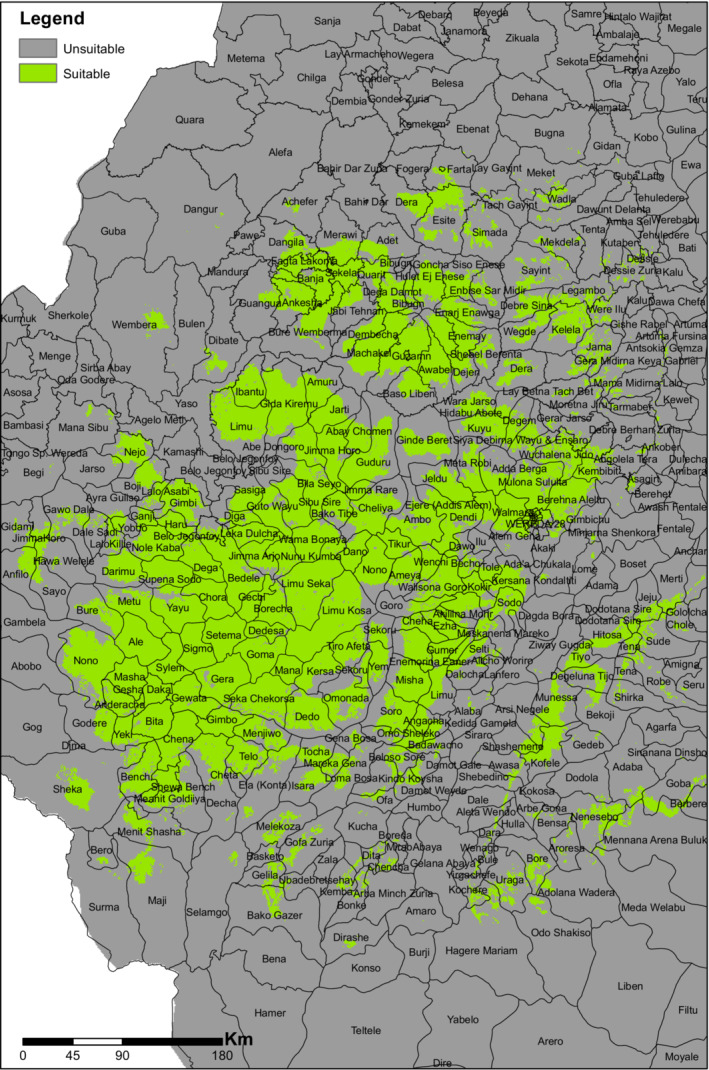
Local districts where habitat suitability is predicted with high certainty.

From the 10th percentile threshold map (Figure 4), we identified 139,378.54 km^2^ and 207,684.49 km^2^ as the suitable geographical distribution of *E. kebericho* at regularization multipliers 1 and 8, respectively. We identified 145,866.14 km^2^ and 131,343.49 km^2^ as representing the suitable geographical distribution for *E. kebericho* at regularization multipliers 1 and 8, respectively, using the maximum training sensitivity plus specificity threshold map (Figure 4). Similarly, using maximum test sensitivity plus specificity, we identified 137,506.21 km^2^ and 180,159.56 km^2^ of the Ethiopian territory as having a suitable geographical distribution for *E. kebericho* at regularization multipliers 1 and 8, respectively (Figure 4). Assembling the maps showed a total of 132,894.574 km^2^ of land in Ethiopia is found to be suitable with high certainty (Figure 5). Areas predicted with high certainty are mainly located on the western Ethiopian massifs (Figures 5 and 6).

## 4 DISCUSSION

This is the first study to look at the potential suitable habitats of the highly important medicinal plant, *E. kebericho*, which is known for its efficacy to cure several human ailments in Ethiopia where health facilities are not fulfilled. Our findings have significant implications for its conservation and management so that the local communities continue to benefit from it.

Our results indicate that the predicted suitable habitats of *E. kebericho* mainly concentrate on the western Ethiopian massifs, while habitat suitability is generally lower on the eastern part except in some mountainous areas (Figure 5). Most of the occurrence data we used for modeling were obtained from the western Ethiopian massifs which is congruent to the predicted suitable areas and the distribution areas of *E. kebericho* described in the Flora of Ethiopia and Eritrea (FEE; Tadesse, 2004). However, the predicted suitable habitats cover much wider areas than the areas that are mentioned in the flora. While *E. kerbricho* is described only from three flora regions, Shoa, Gojam, and Tigray in FEE (Tadesse, 2004), we show that Wallega, Kaffa, Illubabor, Arsi, and Bale highlands are also suitable to *E. kebericho* (Figures 5; Figure S1). This shows there is a mismatch between the actual and potential distributions of *E. kebericho* which is of conservation importance. Particularly high suitability within the western Ethiopian massif confirms the significance of the area to conserve this versatile medicinal plant.

The models run at both complexity levels had high performances and also showed less variations from one run to the other. For species with small sample size or small number of occurrence points, simple models are recommended (Merow et al., 2014). We had minor deviation in AUC values for both simple and complex models, standard deviation that ranges from 0.04 to 0.046. Even though the disproportion between the occurrence and pseudo‐absence data may exaggerate model performances to some degree, the lack of variations in performance across model runs and model complexity levels shows the reliability of the occurrence data we used and the robustness of the models to certain degree. In addition to these, the differences among the binary maps and the uncertainty in the final assembled map were also minor. If the lack of proportion between the two datasets may result in model overfitting, that will lead the models to confine suitable habitats to areas where conditions at presence points are strictly fulfilled, undermining the geographic extent of the predicted suitable habitats. This might be the reason why the predicted suitable habitats are mainly confined to the western Ethiopia massifs, the area where our occurrence localities are concentrating and *E. kebericho* is known to grow. This means, with improved data availability, there is a potential to capture even larger suitable habitats. Better data can also allow to forecast climate change response as well and to use multiple algorithms by applying ensemble modeling approach.

Species may use different strategies to adapt to future climate changes through phenological or physiological modification and in situ adaptation to climate. Species with a diverse habitat can benefit from changing conditions due to distinct phenological or physiological characteristics (Abolmaali et al., 2018). However, species with a narrow geographical distribution are more vulnerable. *Echinops kebericho* is confined to Ethiopian highlands, to elevation ranging from 1700 to 2900 m (Tadesse, 2004). This shows how it is narrowly distributed and vulnerable to changes as well as the importance of introducing it to all potential suitable areas.

Commercial harvesting and sale of *E. kebericho* roots has impacted local populations and will reduce their adaptability to climate change. This could cause the extinction of overused plants in current and future suitable habitats (Svenning et al., 2009). Species distribution models predict the species' fundamental niche besides the realized niche (Kumar & Stohlgren, 2009; Pearson et al., 2007; Yang et al., 2013). In reality, a species might have failed to disperse due to geographical barriers, human disturbance, or associated competitive species (Yang et al., 2013). *Echniops kebericho* has economic and medicinal values and is exposed to dual pressure due to human disturbance (e.g., loss of habitat due to changes in land use and land cover change, and exploitation due to its known medicinal use). Similarly, Ref. Manahlie & Feyissa (2014) reported that *E. kebericho* was a severely endangered medicinal plant mainly due to commercial harvesting and selling of roots. Furthermore, research done in West Gojam Ethiopia (Chandrodyam, 2016) found that *E. kebericho* was on the verge of extinction as a result of overharvesting of the root for medicinal purposes. Those are some of the reasons why we relied on climatic and topographic variables as predictors and tried to capture the potential niche of *E. kebericho* that is masked by anthropogenic and other factors for further conservation efforts.

### 4.1 Conservation strategies *E. kebericho*

The application of results from this research is used in the management and conservation of *E. kebericho*, such as identifying geographical districts for introduction into its suitable range. According to IUCN/SSC (2013), species with small populations, in demographic regression or with high extinction probability have priority when deciding for translocation. In *E. kebericho*, the small population size, habitat fragmentation, and overexploitation would lead to extinction if conservation strategy is not well designed and implemented. Several geographical areas predicted to have suitable habitat for *E. kebericho* may already be devoid of population due to land use change, land cover change, and overexploitation for medicinal and economic purposes. A study by Midgley et al. (2003) proved that the combined effect of future land use, land cover change, and climate change will increase unsuitable habitats in the cape floristic region.

According to the findings of this study, much of the suitable habitat for this plant in the country has already been converted to agricultural land. Likewise, research conducted in Ethiopia's Guraghe zone revealed that agricultural expansion threatens 66% of *E. kebericho* population density (Kloos et al., 2014). In addition to pointing to regions where the species will also find suitable habitats under current climates, our study also identified potential key sites that can support re‐introduction of the *E. kebercho* outside of its current distribution range. Successful conservation planning and strategy for human‐dominated landscapes can be used to address this issue (Urbina‐Cardona & Flores‐Villela, 2010). In response to the loss of suitable habitats, botanical gardens and field gene banks should be established, and *E. kebercho* should be transplanted for cultivation, genetic resource conservation, and sustainable use. Therefore, the Ethiopian Biodiversity Institute, research institutes, and non‐government organizations working on biodiversity conservation should focus on conservation actions such as suitable area protection, species management (species recovery and propagation), education, and awareness‐creation for small‐holder farmers and relevant stakeholders to reduce the risk of *E. kebericho* extinction. To enable the *E. kebricho* occupy the current suitable habitat in Ethiopia, the national green legacy should take the deliberate reintroduction of endangered and nearly extinct medicinal plant species into consideration.

## 5 CONCLUSION

In this study, we predicted the suitable habitats of *E. kebericho* with high AUC values. Our results did not vary across model runs, model complexity levels, and cutting thresholds. Our results indicate that the suitable habitats of *E. kebericho* concentrates on the western Ethiopia highland massifs. Though *E. kebericho* is described only from three flora regions of Ethiopia, we showed that its suitable habitats exist in other flora regions as well and *E. kebericho* has much wider potential distribution. We also mapped these potential distribution areas at local district levels. We recommend the importance of introducing *E. kebericho* to those suitable areas to ensure its conservation as well as to benefit indigenous people who are relying on it for herbal medicines and household income. We hypothesize that other locally confined medicinal plants of Ethiopia may also have wider potential distribution and we recommend a similar approach to ensure their conservation for local and global benefits. However, the high AUC values in our models may attribute to model overfitting and that may undermine the extent of the predicted suitable habitat range. With improved data, wider niche and extent can be mapped. Improved data can also allow assessing climate change impacts and thus highly recommended.

## AUTHOR CONTRIBUTIONS

**Bedilu Tafesse:** Conceptualization (equal); data curation (lead); formal analysis (lead); methodology (equal); software (lead); writing – original draft (lead). **Tamirat Bekele:** Supervision (equal); validation (equal); writing – review and editing (equal). **Sebsebe Demissew:** Supervision (equal); validation (equal); writing – review and editing (equal). **Bikila Warkineh Dullo:** Supervision (equal); validation (equal); writing – review and editing (equal). **Sileshi Nemomissa:** Supervision (equal); validation (equal); writing – review and editing (equal). **Desalegn Chala:** Formal analysis (equal); methodology (equal); software (equal); supervision (equal); writing – review and editing (lead).

## FUNDING INFORMATION

Ababa University, College of Natural Sciences, Department of Plant Biology and Biodiversity Management.

## Supporting information


Figure S1

Table S1

Table S2

## Data Availability

The datasets used in this study are freely available from the following sources: Presence records of the 49 studied species are given in Table S2. Bioclimatic data: WorldClim (https://www.worldclim.org).
