# Supplementary figures and images for: Conservation implications of mapping the potential distribution of an Ethiopian endemic versatile medicinal plant, Echinops kebericho Mesfin
Source: Ecol Evol. 2023 May 7;13(5):e10061. doi: 10.1002/ece3.10061 (PMC10164648; doi:10.1002/ece3.10061)

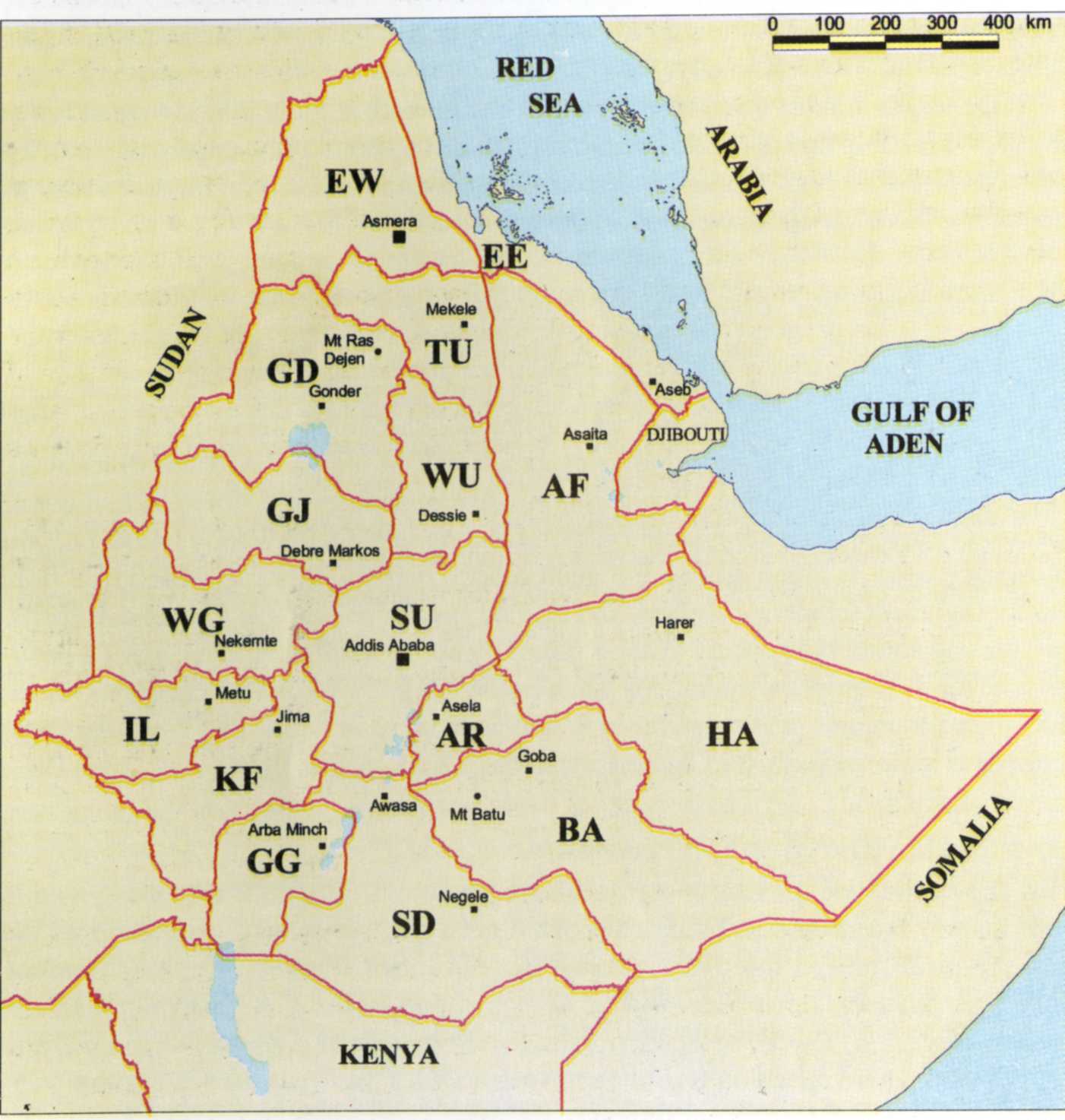


Figure S1. Floristic region of Ethiopia and Eritrea

Supplement: Supplementary file 1 — Figure S1 [file ECE3-13-e10061-s002.docx]

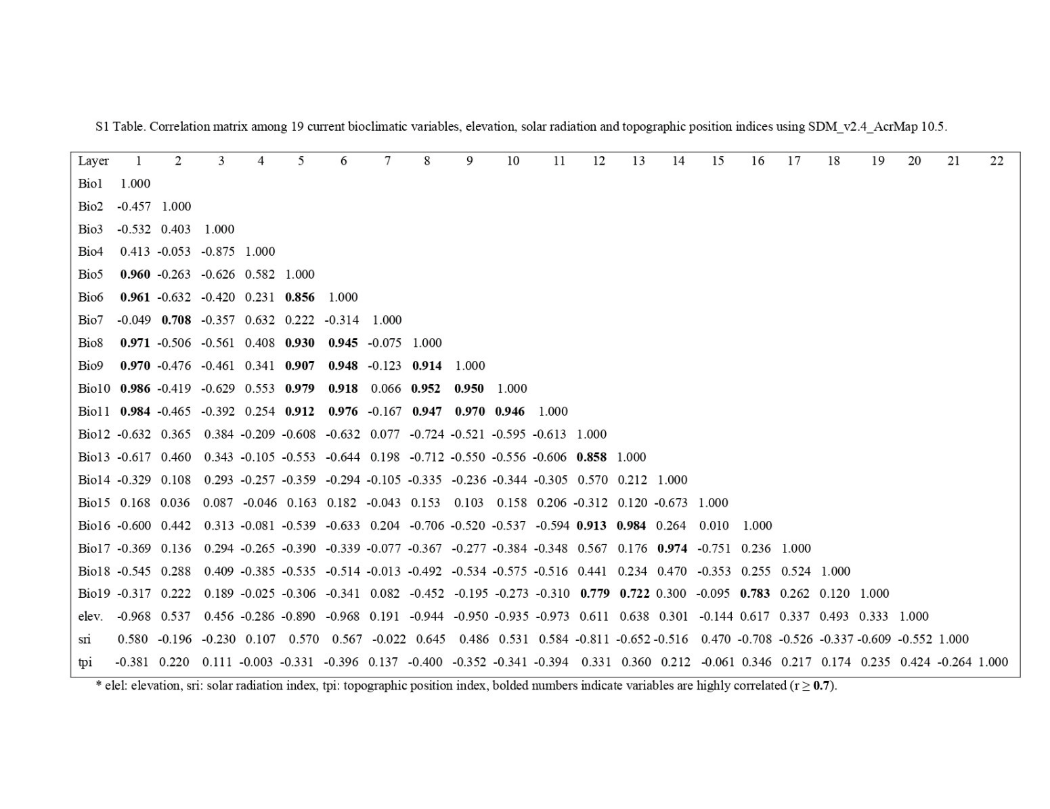
Table S1 correlation matrix among environmental variables

Supplement: Supplementary file 2 — Table S1 [file ECE3-13-e10061-s003.docx]
