# Supplementary material for: Conservation implications of mapping the potential distribution of an Ethiopian endemic versatile medicinal plant, Echinops kebericho Mesfin
Source: Ecol Evol. 2023 May 7;13(5):e10061. doi: 10.1002/ece3.10061 (PMC10164648; doi:10.1002/ece3.10061)
Supplement: Supplementary file 3 — Table S2 [file ECE3-13-e10061-s001.docx]

Table S2 – occurrence points used for modelling and their sources

| No | Latitude | Longitude | Source |
| --- | --- | --- | --- |
| 1 | 9.341251 | 38.9352 | The National Herbarium ETH |
| 2 | 10.561525 | 37.42708 | The National Herbarium ETH |
| 3 | 13.380426 | 39.17283 | The National Herbarium ETH |
| 4 | 9.048326 | 37.89019 | The National Herbarium ETH |
| 5 | 9.03754 | 38.54246 | The National Herbarium ETH |
| 6 | 10.40089 | 37.50084 | The National Herbarium ETH |
| 7 | 9.086504 | 38.50597 | The National Herbarium ETH |
| 8 | 7.158057 | 36.05564 | The National Herbarium ETH |
| 9 | 7.339515 | 36.34885 | The National Herbarium ETH |
| 10 | 9.411044 | 37.44493 | The National Herbarium ETH |
| 11 | 8.986401 | 37.53267 | The National Herbarium ETH |
| 12 | 9.026475 | 37.36483 | Own collection |
| 13 | 8.583338 | 34.80536 | Own collection |
| 14 | 9.783483 | 38.79967 | Own collection |
| 15 | 9.10709 | 38.73576 | Own collection |
| 16 | 9.259402 | 38.73709 | Own collection |
| 17 | 9.319044 | 38.71246 | Own collection |
| 18 | 8.314607 | 36.39492 | Own collection |
| 19 | 7.015156 | 38.15433 | Own collection |
| 20 | 7.102313 | 38.22308 | Own collection |
| 21 | 7.331979 | 37.77715 | Own collection |
| 22 | 9.884746 | 37.09175 | Own collection |
| 23 | 9.840991 | 37.15748 | Own collection |
| 24 | 9.868537 | 37.18844 | Own collection |
| 25 | 7.293477 | 37.61389 | Own collection |
| 26 | 7.279967 | 37.73102 | Own collection |
| 27 | 9.018333 | 36.61519 | Own collection |
| 28 | 9.025789 | 36.70359 | Own collection |
| 29 | 11.051063 | 37.0009 | Own collection |
| 30 | 9.197414 | 35.95716 | Own collection |
| 31 | 9.017664 | 38.35363 | Own collection |
| 32 | 10.313018 | 37.69501 | Own collection |
| 33 | 9.65692 | 37.34275 | Own collection |
| 34 | 9.659832 | 37.14111 | Own collection |
| 35 | 6.943056 | 38.09161 | Own collection |
| 36 | 8.800784 | 37.45067 | Own collection |
| 37 | 9.080803 | 38.15294 | Own collection |
| 38 | 7.44545 | 36.14704 | Own collection |
| 39 | 9.59 | 37.355 | Own collection |
| 40 | 8.131582 | 35.53954 | Own collection |
| 41 | 8.131794 | 35.53866 | Own collection |
| 42 | 8.067145 | 35.61742 | Own collection |
| 43 | 8.204484 | 35.67571 | Own collection |
| 44 | 7.909622 | 35.61728 | Own collection |
| 45 | 7.920545 | 35.57702 | Own collection |
| 46 | 8.283525 | 36.56667 | Own collection |
| 47 | 7.599738 | 35.764181 | GBIF |
| 48 | 10.226667 | 36.986667 | GBIF |
| 49 | 10.211667 | 36.988167 | GBIF |
